# Cell Origami: Self-Folding of Three-Dimensional Cell-Laden Microstructures Driven by Cell Traction Force

**DOI:** 10.1371/journal.pone.0051085

**Published:** 2012-12-12

**Authors:** Kaori Kuribayashi-Shigetomi, Hiroaki Onoe, Shoji Takeuchi

**Affiliations:** 1 Institute of Industrial Science (IIS), The University of Tokyo, Tokyo, Japan; 2 Takeuchi Biohybrid Innovation Project, Exploratory Research for Advanced Technology (ERATO), Japan Science and Technology Agency (JST), Tokyo, Japan; Texas A&M University, United States of America

## Abstract

This paper describes a method of generating three-dimensional (3D) cell-laden microstructures by applying the principle of origami folding technique and cell traction force (CTF). We harness the CTF as a biological driving force to fold the microstructures. Cells stretch and adhere across multiple microplates. Upon detaching the microplates from a substrate, CTF causes the plates to lift and fold according to a prescribed pattern. This self-folding technique using cells is highly biocompatible and does not involve special material requirements for the microplates and hinges to induce folding. We successfully produced various 3D cell-laden microstructures by just changing the geometry of the patterned 2D plates. We also achieved mass-production of the 3D cell-laden microstructures without causing damage to the cells. We believe that our methods will be useful for biotechnology applications that require analysis of cells in 3D configurations and for self-assembly of cell-based micro-medical devices.

## Introduction

*Origami*, the traditional Japanese art of paper folding, has remained popular over the centuries because it enables the production of various three-dimensional (3D) sculptures simply by folding two-dimensional (2D) sheets. In recent years, structural engineers and bio-engineers have been inspired to harness these origami folding techniques for a range of technological applications, including the fabrication of solar panels for space deployment [1], [2], flexible medical stents [3], and nanoscale DNA-based objects [4], [5], leading to the development of a new discipline, “origami engineering” [6], [7].

In the area of microfabrication, origami folding strategies have also proved to be promising approaches for producing 3D microstructures [8]–[14] since they are simple and time-effective compared to other 3D microfabrication techniques such as stereolithography and laser micromachining. In particular, the origami folding techniques have recently been explored to produce various 3D cell-laden microstructures including micro-sized containers [15]–[21] and scaffolds for artificial tissues [22], [23]. The folding of these microstructures is typically performed by surface tension [15], [17], stress-induced forces [16], [21]–[23], and shrinkage of the hinges [18], [19] with external triggers such as temperature and electrical/chemical signals. However, such driving forces require functional materials (e.g. Cu/Cr composite metals [16], [21]–[23] and thermo-sensitive polymers [17]–[19]) that involve complicated preparation processes. In addition, the compatibility of the external triggers to living cells must be considered in these folding mechanisms.

**Figure 1:**
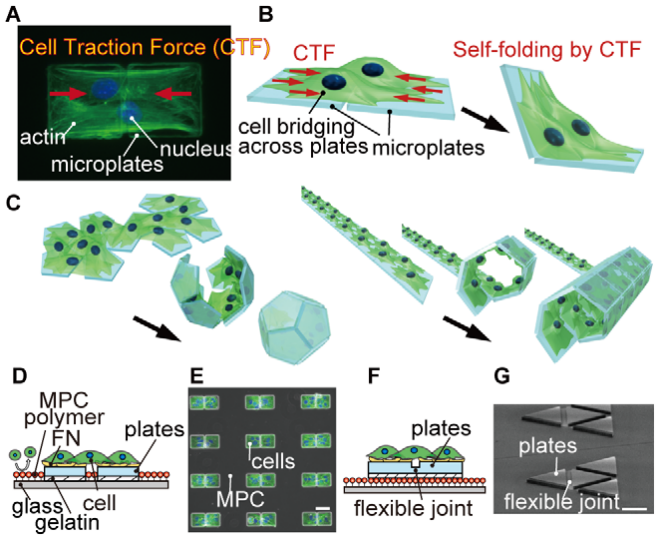
Conceptual illustration of cell origami to produce 3D cell-laden microstructures. (A) The cells adhere and stretch across two microplates, and CTFs are generated toward the center of the cell body. Green and blue colors show actin and nucleus, respectively. (B and C) Schematic image of the cell origami: (B) the cells are cultured on micro-fabricated parylene microplates. The plates are self-folded by CTF. (C) Various 3D cell-laden microstructures can be produced by changing the geometry of the plates. (D) Schematic of the parylene microplates without a flexible joint. The cells are seeded onto the microplates coated with FN. Unwanted cells do not adhere on the glass substrate because of MPC polymer coating. (E) A fluorescent image merged with phase contrast image of NIH/3T3 cells patterned only on the microplates. The cells are bridged across the microplates. (F) Schematic of the parylene microplates with a flexible joint to achieve precise 3D configurations after folding. (G) A SEM image of the microplates with the flexible joint. Scale bars, 50 µm.

In this research, we harness living cells as the self-folding driving forces to create diverse range of 3D cell-laden microstructures: this technique is named *cell origami*. Cells naturally exert a contractile force [24], known as the cell traction force (CTF), that is generated by actomyosin interactions and actin polymerization, and pulls toward the center of the cell body (Figure 1A). The CTF plays a vital role in many biological processes including cell migration, proliferation, and differentiation. Here, we use the CTF to fold 2D microstructures by patterning cells across a pair of microplates and detaching the microplates from the glass substrate (Figure 1B). Cell origami is highly biocompatible and does not require any special materials for the microplates and hinges to induce folding. In addition, we can produce various 3D cell-laden microstructures by just changing the geometrical design of the patterned 2D plates (Figure 1C).

## Results and Discussion

### Culturing cells across microplates

We examined the basic mechanism and design criteria of our cell origami by culturing cells on a set of two microplates that are put side by side to form a single folded microstructure. We applied two types of cell origami: microplates with and without a flexible joint (Figures 1D and F, Figures S1 and S2). The detail of the microplate preparation steps is described in the Materials and Methods section. In both cases, selective patterning of the cells on the microplates was achieved by coating the glass substrate areas, where the microplates do not exist, with 2-methacryloyloxyethyl phosphorylcholine (MPC) polymer; this polymer inhibits protein adsorption and cell adhesion [25]. As a microplate, we choose a micro-patterned parylene (poly(p-xylylene) polymer) film coated with fibronectin (FN). It is known that the effect of the material properties of the parylene coated with FN on CTF, including cell stretching and cell spreading, is comparable to that of standard tissue culture substrates such as polystyrene [26]. Thus, parylene coated with FN can be a suitable material for culturing cells. Between the microplates and the glass substrate, there is a sacrificial gelatin layer in order to allow detachment of the microplates from the substrate. The critical conditions for using the CTF as the self-folding driving force include: (i) the concentration of cells; (ii) the distance between the two microplates; and (iii) the adherence of the sacrificial gelatin layer under the microplates.

**Figure 2:**
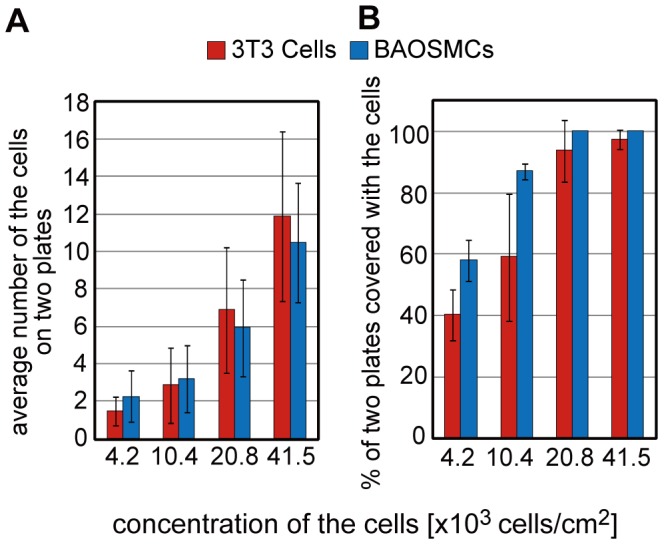
Condition of culturing cells across a set of two microplates. (A) The number of cells on a set of two microplates is reduced by decreasing the concentration of the seeded cells. (B) The proportion of a set of two microplates covered by cells increases as the concentration of cells increases. Size of the microplates is 50 µm×50 µm. Results are shown as the mean ± s.d. (a: *n* = 3–5: 100 samples were observed each experiment, b: *n* = 100–282).

Cells were successfully patterned onto the parylene microplates coated with FN, and they adhered and stretched onto the plates (Figure 1E). The number of patterned cells on the microplates was controlled by the concentration of the seeded cells (Figure 2A). The proportion of a set of two microplates covered by cells increased as the concentration of cells increased (Figure 2B). Therefore, from the results in Figures 2A and B, we found that we are able to pattern cells at single cell level on ∼40% (NIH/3T3 cells) and ∼60% (bovine aortic smooth muscle cells: BAOSMCs) pairs of the microplates, and to pattern multiple cells (6–10 cells) on almost 100% pairs of the microplates for both types of cells.

**Figure 3:**
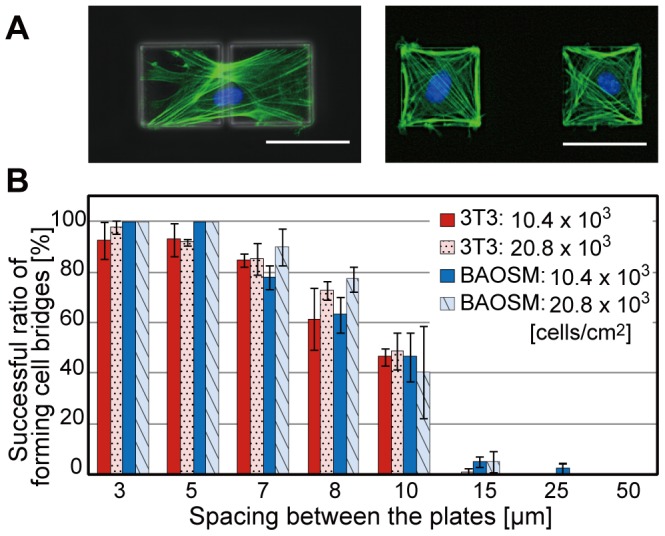
Construction of a cell bridge between a set of two microplates. (A) Fluorescent images merged with phase contrast images of BAOSMCs on a set of two microplates with spacing of 5 µm (top image) and 50 µm (bottom image). The actin filaments and nuclei of the patterned cells on the microplates are fluorescently stained green and blue, respectively. (B) A graph of the ratio of successfully-formed cells bridge between the two microplates against various plate spacing, different concentrations of the cultured cells, and two different cell types. Results are shown as the mean ± s.d. (*n* = 3–10: 100 samples were observed each experiment). Scale bars, 50 µm.

After the cells were patterned, they extended their filopodia and bridged across pairs of the microplates. The spacing between the plates is a critical criterion that determines whether the cells can bridge them in order to fold the microplates by the CTF (Figure 3A). We found that about 80% of the cells could bridge microplates with spacing less than 7 µm (Figure 3B). Most of the cells, however, could not bridge when the spacing was more than 15 µm. Therefore, the spacing between the plates should be less than 7 µm to produce the cell origami.

### Folding of the microplates by CTF

We experimentally investigated how the cells folded from 2D microplates into 3D microstructures. First, we cultured the cells on a set of two microplates without a flexible joint for 24–48 h. The microplates were then slightly pushed at their edges with a glass tip operated by a micromanipulator in order to trigger detachment of the microplates from the glass substrate (Figure S3, Movie S1). After the trigger, the detached microplate was pulled by the CTF generated by the cells cultured on the microplates until it contacted the other microplate. Since the CTF acted only on the upper surfaces of the plates, a rotational movement was generated at the contacted upper edge of the microplate, lifting the microplate off from the glass substrate.

When multiple cells are patterned on a set of two microplates, the cells contact each other. In this case, the main driving force that folds the microplates is the CTF exerted by cell-matrix interaction at the cells' periphery; CTFs exerted by multiple cells are directed centripetally at the cells' periphery, and no large traction stresses are exerted under sites of cell-cell contact [27].

In addition, the concentration of gelatin of the sacrificial layer between the parylene microplates and the glass substrate is important to successful folding. We found that when 0.05–0.1% gelatin was used, the microplates were retained on the substrate during culturing, so that the cells were able to fully stretch across the microplates. The microplates could then be selectively released after the triggering. When the concentration of the gelatin was higher than 0.1%, the microplates frequently detached from the glass substrate without the trigger during culturing due to the CTF, resulting in dragging and overlapping of the microplates instead of proper folding. When the concentration of the gelatin was lower than 0.05%, the microplates could not detach.

### Characterization of the folding angle

**Figure 4:**
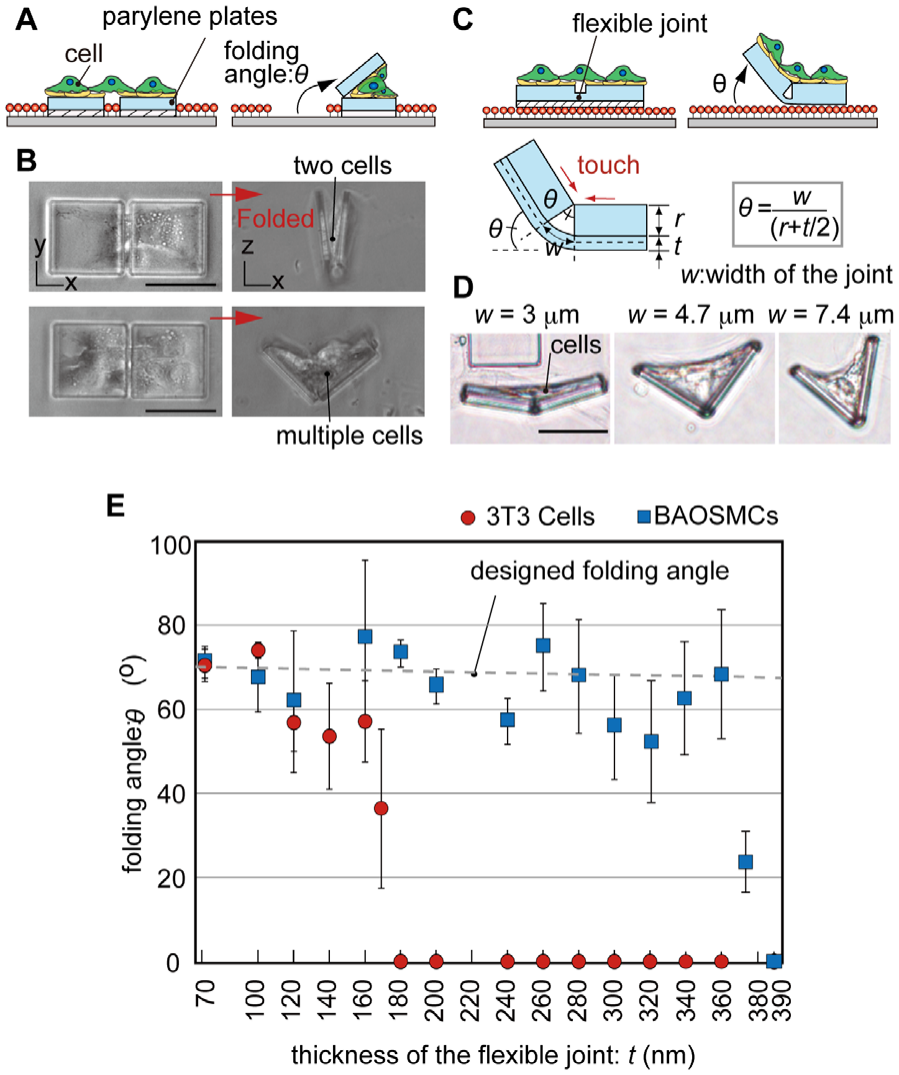
Characterization of the folding angles. (A) Schematic illustration of folding parylene microplates without a flexible joint. The plates are folded until the microplates are blocked by the cells. (B) Phase contrast images before and after folding of the microplates without the joint having different cell density of NIH/3T3 cells. (C) Schematic illustration of folding microplates with a flexible joint. The folding angle, *θ*, is defined as the angle between the folded microplates and the glass substrate. The plates are folded until the edges of the plates contact each other. (D) Phase contrast images after folding parylene microplates with different *w* of the flexible joint. Different *θ* are achieved by changing the value of *w* using BAOSMCs. (E) The relationship between *θ* and *t* for NIH/3T3 cells and BAOSMCs when *w* = 4.68 µm, *w* = 3.8 µm. Results are shown as the mean ± s.d. (*n* = 3–14: 100 samples were measured each experiment). Scale bars, 50 µm.

An important parameter for producing desired 3D cell-laden microstructures is the folding angle, *θ*, between the folded microplate and the glass substrate (Figure 4A). The folding continues until the microplates are blocked by the cells. Thus, the folding angle can approximately be determined by the number of the cells on the microplates. When two or less cells bridged across two microplates without a flexible joint, the plate folded almost completely (folding angle >160±5°) (Figure 4B). With an increase in the number of cells on the microplates, the microplates were blocked by the multiple cells and could not be folded further, thus the folding angle decreased.

In order to control the folding angle more precisely, we engineered an additional thin and flexible joint between the microplates (Figures 1G, 4C). The microplates are self-folded by the CTF until the top inner edges of the two microplates connected by the flexible joint touch each other (Figure 4C). The folding angle does not depend on the number of cells on the microplates. Instead, it is determined geometrically by the thickness, *t*, and the width, *w*, of the flexible joint, and the thickness of the microplate, *r*, as follows: *θ* = *w*/(*r*+*t*/2). In our experiment, the flexible joint is 3–8 µm in width and 70–390 nm in thickness. We successfully controlled the folding angle using the microplates with the flexible joint. The folding angle increased as the width, *w*, increased (Figure 4D). As the thickness of the joint, *t*, increases, the joint becomes stiffer (stiffness∝*t* ^3^), therefore, the cells cannot fold the flexible joint beyond a certain joint thickness. We found that the joint thickness that allowed folding with a precise angle depended on the types of cells. For example, BAOSMCs and NIH/3T3 cells were able to fold the joints to the designed folding angle when the thicknesses were less than 360 nm and 160 nm, respectively (Figure 4E); BAOSMCs folded thicker joints than NIH/3T3 cells. The cell types must also be considered when designing the microplates with the flexible joint. Behaviors of the folding are also depended on the cell types. In the case when primary rat cardiomyocytes were used, the microplates were folded and deployed continuously with a precise folding angle like “micro-flapping” structures (Movie S2).

### Self-folding of 3D cell-laden microstructures

**Figure 5:**
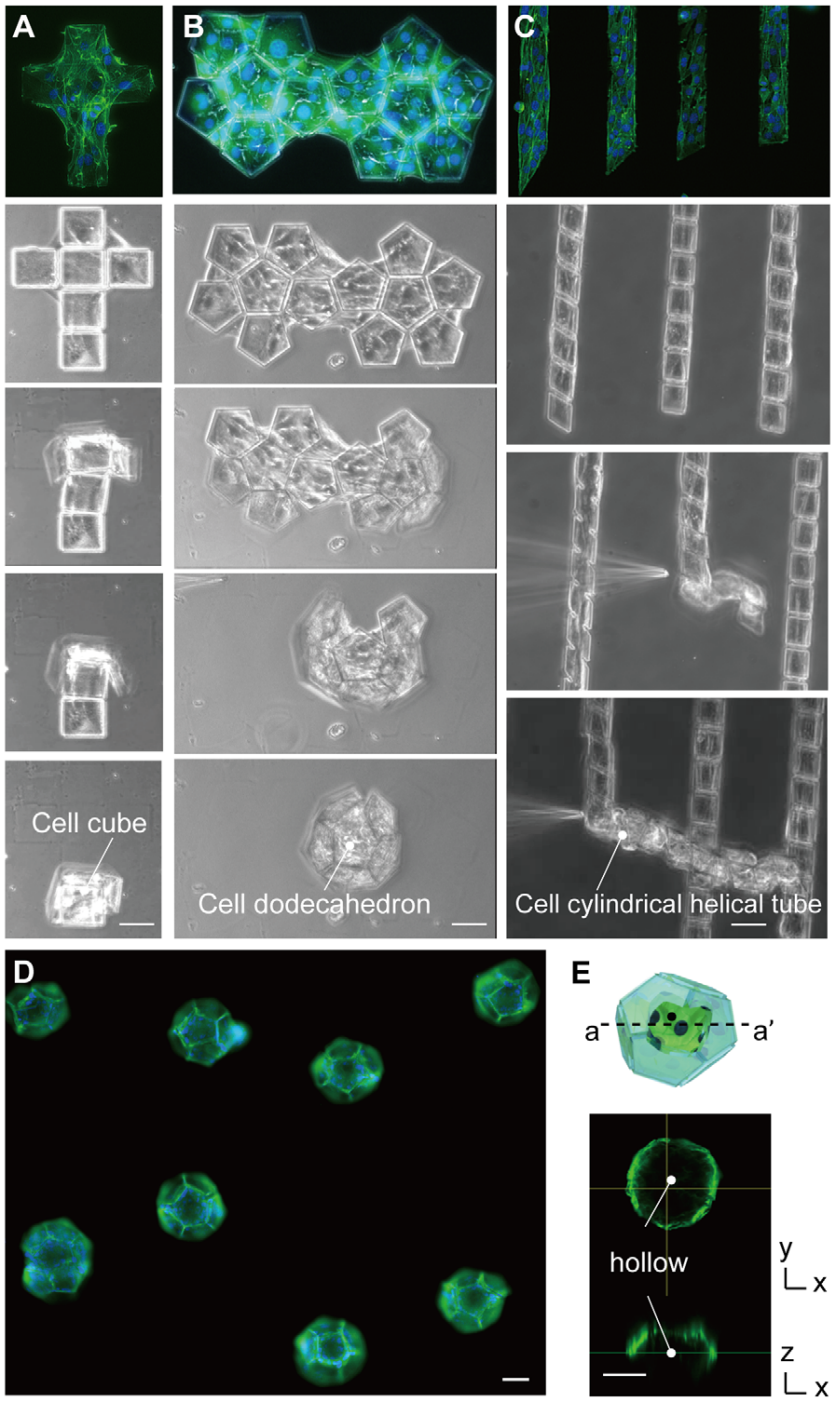
Sequential images of various 3D cell-laden microstructures folded by CTF. (A)–(C) Regular tetragon, regular dodecahedron and cylindrical tube were self-folded. (D) A fluorescent image of regular dodecahedrons. (E) A cross-section (a–a′) image of the dodecahedron structure in a hollow shape taken by a confocal scanning laser microscopy. Green and blue colors show actin and nucleus, respectively. NIH/3T3 cells were used. Scale bars, 50 µm.

Various 3D cell-laden microstructures were produced by using the defined conditions to bridge the cells and controlling the geometry of the patterned 2D microplates. Using the microplates without the flexible joint, we successfully produced three hollow microstructures: a cube, a dodecahedron, and a cylindrical helical tube (Figure 5A–C; Movies S3, S4, S5) with NIH/3T3 cells. In the case of the cylindrical tube, the microplates rolled up along diagonal lines. The tube diameter can be adjusted by changing the angle of the diagonal lines. We also produced cylindrical tubes using bovine carotid artery endothelial cells and normal human umbilical vein endothelial cells (HUVECs) in order to mimic vessel-like structures (Figure S4). An array of 3D dodecahedrons was also successfully produced, with each dodecahedron being produced one at a time (Figure 5D). We confirmed that the produced dodecahedrons were hollow by imaging with a confocal microscope (Figure 5E).

### Batch process of self-folding 3D cell-laden microstructures

To widen the field of practical applications of the cell origami technique toward cell-laden microstructures, the produced microstructures need to be reproducible and suitable for mass production. For the microplates incorporating the flexible joint with a 3–5% gelatin sacrificial layer, we found that the microplates were self-folded by the CTF without any extra triggers, and parallel processing of self-folding 3D cell-laden microstructures was achieved.

**Figure 6:**
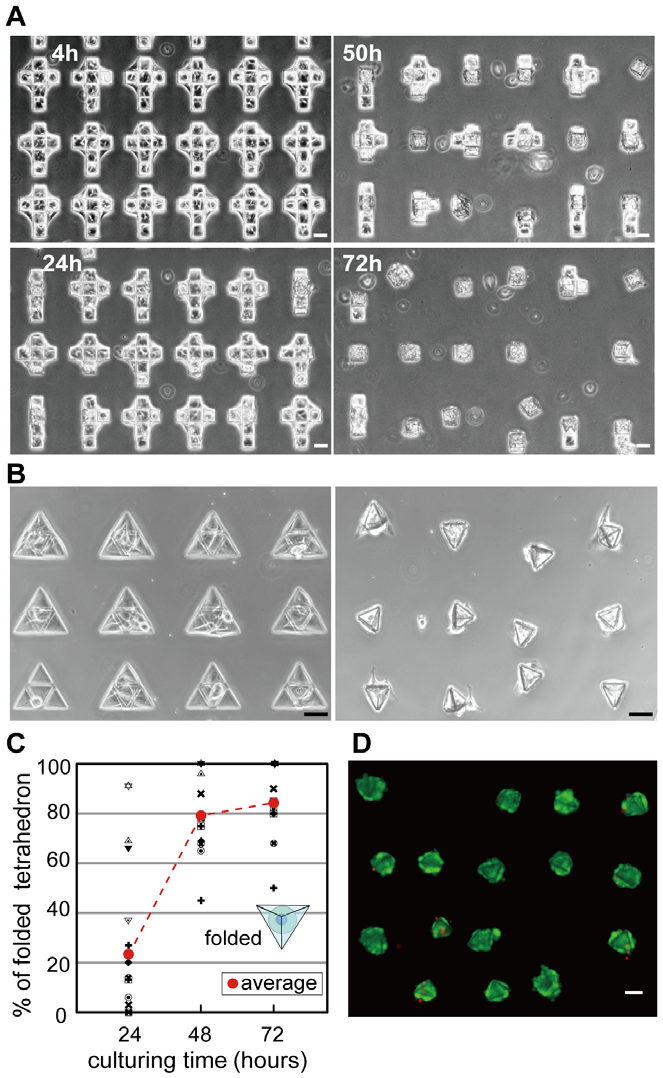
Batch process of folding cells-cultured microplates with flexible joint. (A) Sequential images of batch process of cubes. (B) Batch processing of tetrahedron before and after self-folding. (C) Ratio of the folded tetrahedron vs. culturing time. (D) Fluorescent microscopic image of the cells after culturing the cells for 4 days with the live/dead fluorescent staining. Live and dead cells are shown in green and red colors, respectively. NIH/3T3 cells were used. Scale bars, 50 µm.

Cubic and tetrahedral microstructures were self-folded spontaneously, and as a result the 3D cell-laden microstructures were successfully mass-produced (Figures 6A, B, Movie S6). We also achieved batch-process of the 3D cell-laden microstructures at a typical density of 1200 structures/cm^2^ with 84.44% folding success rate within 3 days after seeding the cells onto the microplates (Figure 6C). We found that most cells were viable after folding, as determined by fluorescent imaging using LIVE/DEAD® Viability/Cytotoxicity kit (Figure 6D). The cells inside the microstructure were also viable after culturing the cells for 7 days as confirmed by imaging with a confocal microscope (Figure S5).

## Conclusions

In this study, we exploit the CTF to drive the folding of 2D microplates into 3D cell-laden microstructures. Cell origami is a highly biocompatible, simple, and efficient technique with a single step to encapsulate cells into the microstructures. It is particularly useful for producing hollow structures with cells in various shapes including cylindrical tubes and cubes. Therefore, this technique is suitable for fabricating artificial tissues in hollow shapes and next-generation cell-based biohybrid medical devices such as stents/grafts, and for realizing advancements in basic cell biology studies under flexible and configurable 3D environments [28]–[30].

## Materials and Methods

### Preparation of a substrate with parylene microplates and MPC polymer

We mainly used parylene C (Specialty Coating Systems, USA) to produce the microplates because it offers several advantages including ease of microfabrication and biocompatibility [26]. In addition, it is transparent, thus allowing observation of the assembly of the 3D cell-laden microstructures under a microscope. Furthermore, free-standing parylene microplates are sufficiently stiff to prevent wrinkling under the CTF during cell growth.

Figure S1 shows the process flow of producing the microplates without a flexible joint and culturing the cells on the plates. We produced 3–4-µm-thick parylene microplates (Figure S1A–i). In detail, the parylene was deposited by chemical vapor deposition (CVD) with a parylene deposition machine (LABCOTER PDS2010, Specialty Coating Systems, USA) on a glass substrate spin-coated with 0.05–0.1% gelatin (Sigma-Aldrich, USA) at 2000 rpm. The gelatin can be dissolved at 37°C, therefore, it serves as a sacrificial layer that enable the microplates to release from the substrate when the plate is folded by CTF. The parylene film was then etched away with O_2_ plasma (10 ml/min, 25 W) (RIE-10NR, SAMCO, Japan) at the defined regions with aluminum (Al) mask that was patterned using standard photolithographic technique (Figure S1A–ii). Before removing the Al, we coated the glass substrate with MPC polymer to inhibit protein adsorption and cell adhesion (Figure S1A–iii). Specifically, MPC solution was spun at 2000 rpm for 30 s onto the substrate, and then the substrate was dried in a chamber with an ethanol atmosphere at room temperature for 20 min to form the polymer layer uniformly. The substrate was then baked at 70°C for 4 h to covalently graft the MPC polymer to the surface of the exposed glass area in the substrate by a dehydration reaction. The Al worked as a sacrificial layer for the MPC layer, and the MPC layer was subsequently lifted-off by removing Al with an alkaline solution (NMD developer, Tokyo Ohka, Japan), leaving behind bare parylene surface (Figure S1A–iv). Therefore, the cells can only be seeded onto the patterned parylene plates that are coated with FN (Funakoshi, Japan) at 10 µg/ml concentration (Figure S1A–v–vi).

Figure S2 shows the process flow of producing the microplates with a flexible joint and culturing the cells on the plates. The first step is forming MPC polymer and gelatin layers on a glass substrate, so that the cells cannot adhere the substrate after the folding. 3–5% gelatin solution was spin-coated. We then deposited a parylene film onto the MPC polymer and gelatin layers (Figure S2–i). In order to produce a flexible joint between the microplates, we patterned a photosensitive polymer SU-8 (Micro Chem, USA) and then deposited the parylene (Figure S2–ii–iii). Next, we etched the parylene film to produce the microplates using O_2_ plasma with an Al mask that was patterned by standard lithography (Figure S2–iv). After that, the MPC polymer was coated again on the glass substrate since the polymer was also etched and adversely affected by O_2_ plasma (Figure S2–v). Finally, the Al mask was removed using NMD, and the microplates with the flexible joint were produced (Figure S2–vi). Figure 1G shows an image of the produced microplates with the flexible joint taken by scanning electron microscopy (SEM) (VHX-D510, Keyence, Japan). When the plates are folded, the joint works as a valley fold.

### Culturing the cells

We used NIH/3T3 cells (TKG299, RIKEN Cell Bank, Japan) and BAOSMCs (CAB35405, Cell Applications) that are commonly used as adherent cells to study CTFs; they spread on a substrate and generate CTFs on its surface [24], [31], [32]. We also used bovine carotid artery endothelial cells (JCRB cell bank, Japan), HUVECs (CC2519, Lonza Walkersville), and primary rat cardiomyocytes (CMC02, Primary Cell, Japan) to explore further variation of cell types to fold 3D microstructures.

The NIH/3T3 cells and bovine carotid artery endothelial cells were cultured in Dulbecco's modified Eagle's medium (DMEM) supplemented with 10% fetal bovine serum (FBS) (JBS-5441, JBS-016549, Japan Bioserum) and 1% penicillin-streptomycin solution (AB) (Japan Bioserum) at 37°C under a humidified atmosphere of 5% CO_2_. In the case of HUVECs and cardiomyocytes, EGM®-2 BulletKit™ (Lonza Walkersville) and CMCM (Primary Cells, Japan) supplemented with 10% FBS, 10 units/ml penicillin, and 10 µg/ml streptomycin were used, respectively. For seeding the cells onto the microplates, the cells were harvested with trypsin (Gibco, Tokyo, Japan) and collected in the culture media containing trypsin inhibitor (Sigma).

We prepared cells suspended in culture media at various concentrations (cells/ml). Then, we added 2 ml of the cell suspension into a petri dish containing our substrate with the microplates. The sizes of the dish and the substrate were 35 mm in diameter and 12 mm×14 mm, respectively. The percentage of the area to be patterned with cells (area sum of microplates) from the whole area (area sum of microplates and areas of the bottom of the dish and of MPC coating) is ca.2%. The cell seeding concentration was 2×10^4^–2×10^5^ cells/ml, which corresponds to 4.2×10^3^–41.5×10^3^ cells/cm^2^. Non-adhered cells on the substrate were washed out after a 4 h culture period (Figures S1a-vi, S2vi).

### Detachment of the microplates

There are two folding approaches. In the first approach, we used 0.05–0.1% gelatin as the sacrificial layer, and the edges of individual microplates were pushed with a glass tip manipulated by a micromanipulator (NI2, Eppendorf), manually triggering detachment of the plates from the substrate (Figures 1D, S1 a-vii, movies S3, S4, S5). Thus, a large amount of the microstructures can be produced in order. In the second approach, we incorporated the flexible joint (Figures 1F, S2viii, movies S2, S6) with a 3–5% gelatin sacrificial layer. In this case, microplates were detached and self-folded by CTF spontaneously as the gelatin dissolved at 37°C, which is the temperature at the cell incubator. Consequently, many 3D microstructures were produced simultaneously. The optimum concentrations of the gelatin in both approaches were experimentally determined (Table S1).

### Cell morphology and cell staining

In order to visualize cell morphology, the cells were first fixed with 4% paraformaldehyde (PFA, Muto Pure Chemicals, Japan) for 15 min and rinsed three times with phosphate buffered saline (PBS, Sigma, USA). The cells were permeabilized with 0.1% TritonX-100 (Sigma, USA) for 2 min and rinsed three times with PBS. In order to avoid non-specific binding, the substrate was immersed into 1% bovine serum albumin (BSA, Sigma, USA) solution for 30 minutes and rinsed once with PBS. The cells were then incubated with Alexa Fluor Phalloidin 488 conjugate (Molecular Probes; 1∶200 dilution) and Hoechst 33342 (Molecular Probes; 1∶400 dilution) to stain their actin filaments and nuclease with green and blue, respectively. After that the cells were rinsed three times with PBS.

We used the fluorescent imaging kit LIVE/DEAD® Viability/Cytotoxicity to determine cell viability (Invitrogen, USA). The staining was performed in accordance with the manufacturer's instructions. The cells were stained just before the image acquisition when microplates have already been folded. All processes were performed at room temperature.

### Imaging equipment

The morphology of the cultured cells on the microplates was observed using an inverted optical microscope with phase contrast (IX71, Olympus, Japan). The images (Figures 4B, D) were captured using a CCD camera (DP72, Olympus, Japan) with an image software (AioVision, Olympus, Japan). Time-lapse images of the self-folding process by CTF with phase contrast were captured with CCD cameras (QICAM, Roper, US) (Figures 5A–C) or (AxioCam HRc, Carl Zeiss, Germany) (Figures 6A, B). To observe the fluorescence images of actin filaments and nucleases, we used an inverted optical fluorescence microscope with CCD camera and imaging software (BZ-9000, Keyence, Japan). The z-stack images of the cell origami (Figure 5E) were taken by a confocal laser scanning microscope (Fluoview FV1000, Olympus, Japan).

## Supporting Information

Movie S1 Time-lapse images of self-folding microstructures with cells across a pair of the microplates by CTF.
(MOV)

Movie S2 Time-lapse images of continuously folding and deploying plates with a flexible joint driven by the **cardiomyocytes** cultured on the plates.
(MOV)

Movie S3 Time-lapse images of self-folding 3D cell-laden structure by CTF: **cube**.
(MOV)

Movie S4 Time-lapse images of self-folding 3D cell-laden structure by CTF: **dodecahedron**.
(MOV)

Movie S5 Time-lapse images of self-folding 3D cell-laden structure by CTF: **cylindrical helical tube**.
(MOV)

Movie S6 Time-lapse images of **batch process of self-folding 3D cell-laden**.
(MOV)

Figure S1 **Schematic illustration of the fabrication steps of self-folding using the microplates.** (A) (i)–(ii) Parylene microplates were produced by using standard photolithography. (iii)–(iv) MPC polymer was coated to prevent cells from adhering the areas without the microplates. (v)– (vii) Cells were cultured onto the microplates coated with FN, and the plates were self-folded by CTF when trigger was applied (Figures 4B and 5 in main text). (B) Culturing the cells onto substrates coated with and without MPC polymer.
(TIF)

Figure S2 **Schematic illustration of the fabrication steps of self-folding using the microplates with a flexible joint.** (i)–(iv) The microplates with the flexible joint were produced with parylene and SU-8 by using standard photolithography. (v)–(vi) MPC polymer was coated to prevent cells from adhering the areas without the microplates. (vii) Cells were cultured onto the microplates, and (viii) the plates were self-folded by CTF spontaneously (Figures 4D and 6 in main text).
(TIF)

Figure S3 **Self-folding mechanism.** The CTFs were in equilibrium between a set of two microplates before detaching the plates from the glass substrate. We then pushed the plates using a glass tip, triggering detachment of the plates from the substrate. The cells pulled the upper faces of the detached plates by the CTFs, dragging the plates towards one another until their edges contact. Although the edges were pushing each other, the CTFs acted only on the upper surfaces of the plates, generating a rotational movement along the contacted upper edge. Consequently, the plates lifted out from the glass substrate and self-folded (Movie S1).
(TIF)

Figure S4 **Images of cylindrical tubes** with (A) bovine carotid artery endothelial cells and (B) HUVECs as vessel-like structures. Scale bars, 50 µm.
(TIF)

Figure S5 **Cross-section images of cells inside the microstructures after culturing the cells for 7 days.** The images of the cells inside the (A) cube and (B) dodecahedron at top (t), middle (m), and bottom (b) taken by a confocal scanning laser microscopy. Live and dead cells are shown in green and red colors, respectively. Scale bars, 50 µm.
(TIF)

Table S1 **Concentrations of gelatin for folding microplates with and without a flexible joint.**
(TIF)
