# Supplementary figures and images for: Cell Origami: Self-Folding of Three-Dimensional Cell-Laden Microstructures Driven by Cell Traction Force
Source: PLoS One. 2012 Dec 12;7(12):e51085. doi: 10.1371/journal.pone.0051085 (PMC3521028; doi:10.1371/journal.pone.0051085)

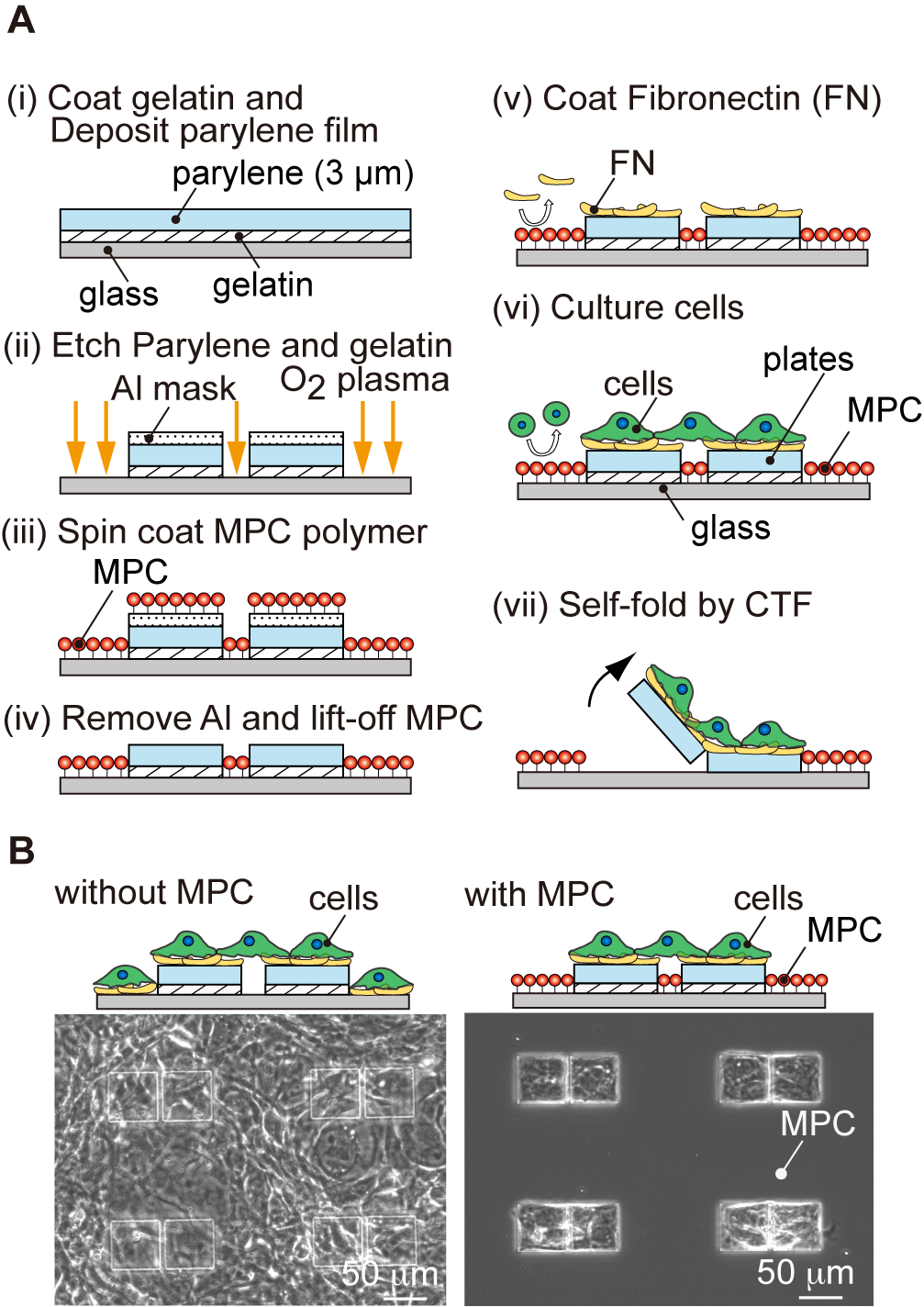

Supplement: Figure S1 — Schematic illustration of the fabrication steps of self-folding using the microplates. (A) (i)–(ii) Parylene microplates were produced by using standard photolithography. (iii)–(iv) MPC polymer was coated to prevent cells from adhering the areas without the microplates. (v)– (vii) Cells were cultured onto the microplates coated with FN, and the plates were self-folded by CTF when trigger was applied (Figures 4B and 5 in main text). (B) Culturing the cells onto substrates coated with and without MPC polymer. (TIF) [file pone.0051085.s007.tif]

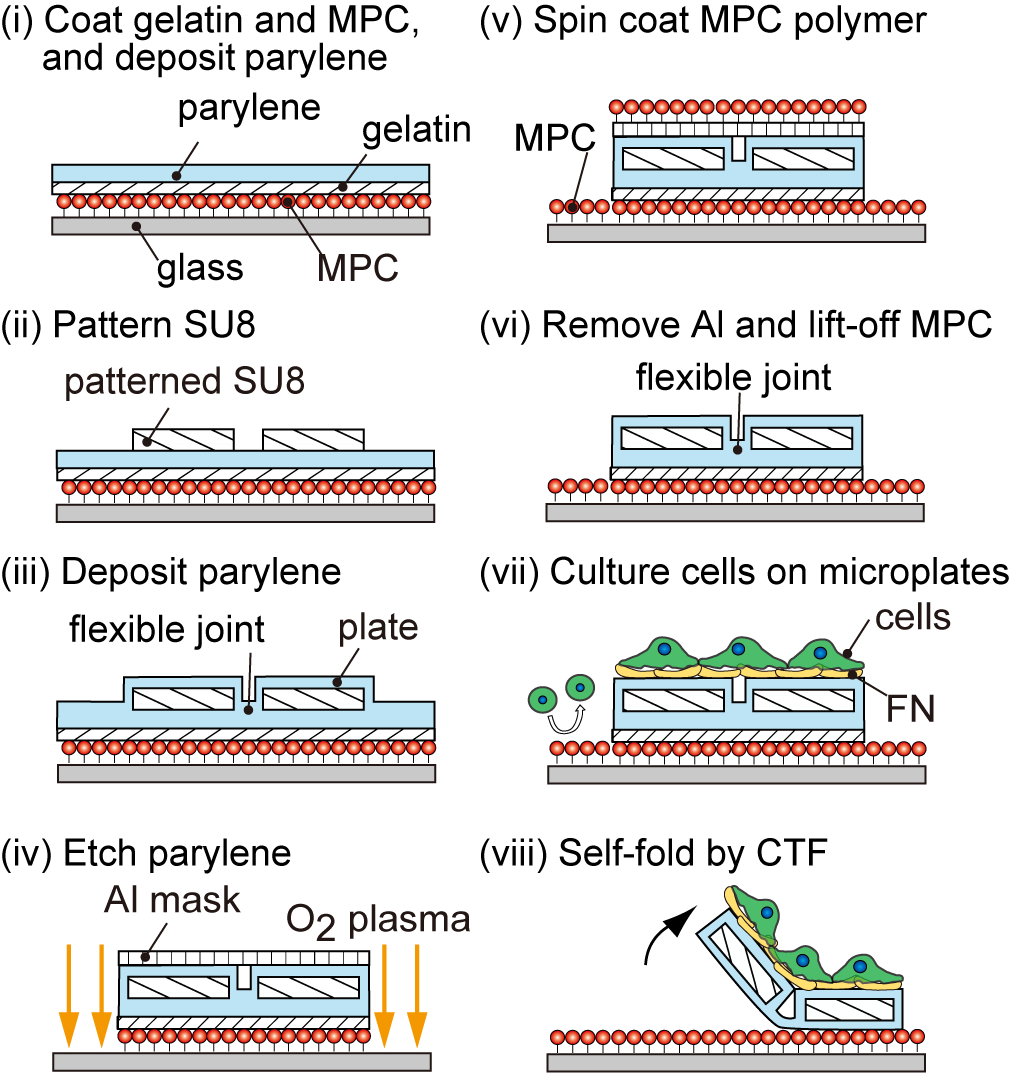

Supplement: Figure S2 — Schematic illustration of the fabrication steps of self-folding using the microplates with a flexible joint. (i)–(iv) The microplates with the flexible joint were produced with parylene and SU-8 by using standard photolithography. (v)–(vi) MPC polymer was coated to prevent cells from adhering the areas without the microplates. (vii) Cells were cultured onto the microplates, and (viii) the plates were self-folded by CTF spontaneously (Figures 4D and 6 in main text). (TIF) [file pone.0051085.s008.tif]

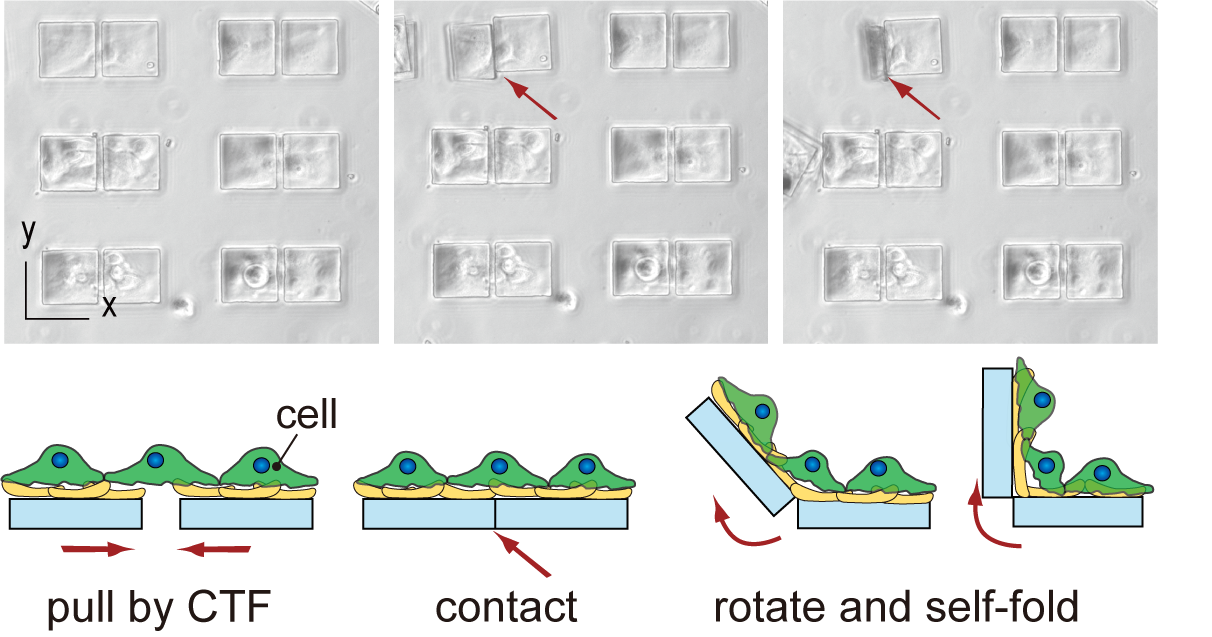

Supplement: Figure S3 — Self-folding mechanism. The CTFs were in equilibrium between a set of two microplates before detaching the plates from the glass substrate. We then pushed the plates using a glass tip, triggering detachment of the plates from the substrate. The cells pulled the upper faces of the detached plates by the CTFs, dragging the plates towards one another until their edges contact. Although the edges were pushing each other, the CTFs acted only on the upper surfaces of the plates, generating a rotational movement along the contacted upper edge. Consequently, the plates lifted out from the glass substrate and self-folded (Movie S1). (TIF) [file pone.0051085.s009.tif]

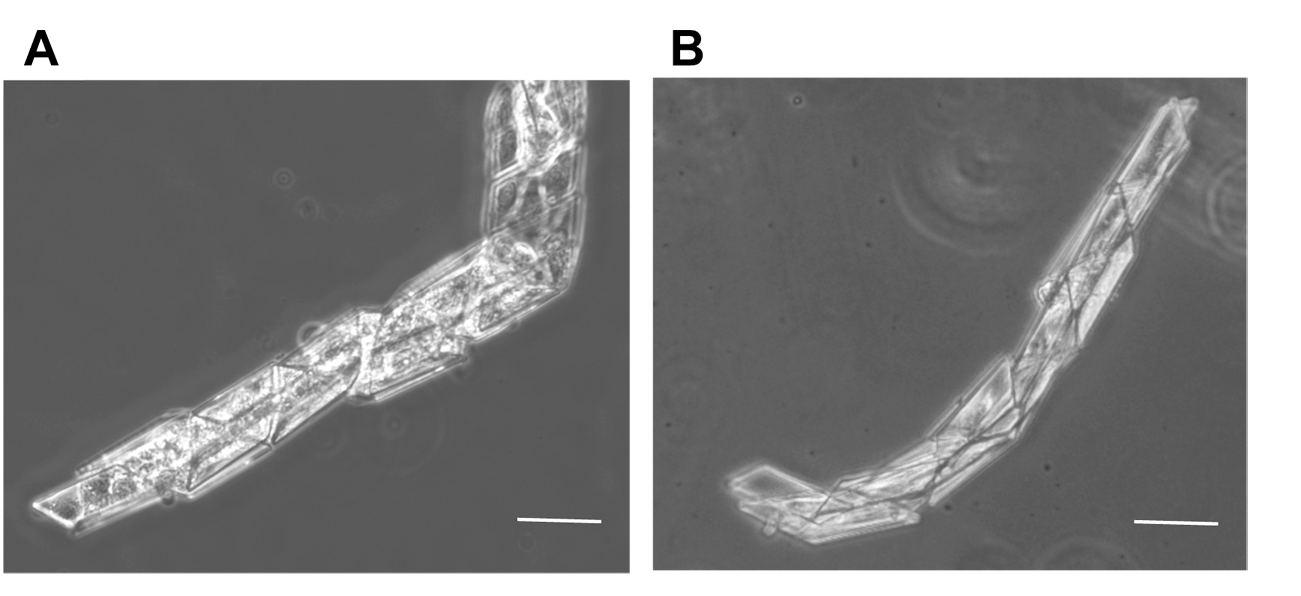

Supplement: Figure S4 — Images of cylindrical tubes with (A) bovine carotid artery endothelial cells and (B) HUVECs as vessel-like structures. Scale bars, 50 µm. (TIF) [file pone.0051085.s010.tif]

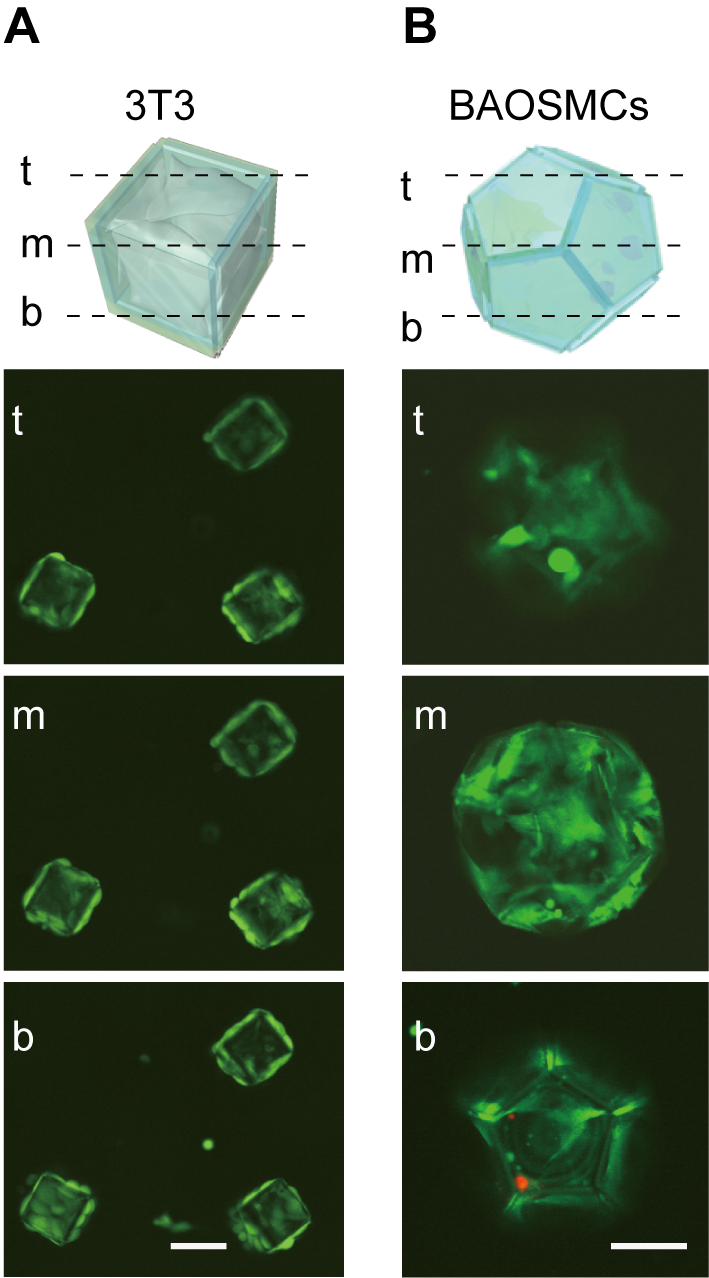

Supplement: Figure S5 — Cross-section images of cells inside the microstructures after culturing the cells for 7 days. The images of the cells inside the (A) cube and (B) dodecahedron at top (t), middle (m), and bottom (b) taken by a confocal scanning laser microscopy. Live and dead cells are shown in green and red colors, respectively. Scale bars, 50 µm. (TIF) [file pone.0051085.s011.tif]

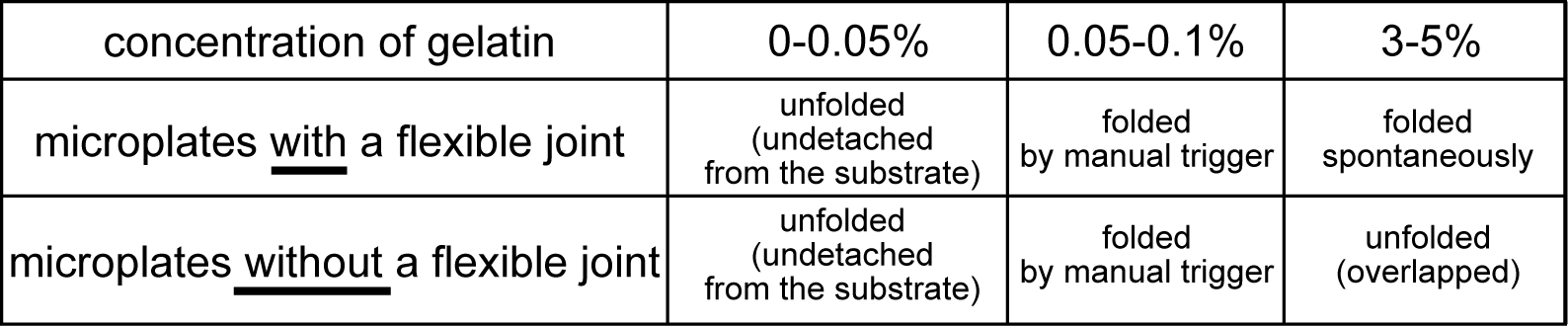

Supplement: Table S1 — Concentrations of gelatin for folding microplates with and without a flexible joint. (TIF) [file pone.0051085.s012.tif]
